# Retinoic acid receptor signaling preserves tendon stem cell characteristics and prevents spontaneous differentiation in vitrox

**DOI:** 10.1186/s13287-016-0306-3

**Published:** 2016-03-22

**Authors:** Stuart Webb, Chase Gabrelow, James Pierce, Edwin Gibb, Jimmy Elliott

**Affiliations:** Genomics Institute of the Novartis Research Foundation, 10675 John Jay Hopkins Drive, San Diego, CA 92121 USA

**Keywords:** Retinoic acid receptor, Scleraxis, Tendon stem cells, Proliferation, Spontaneous differentiation

## Abstract

**Background:**

Previous studies have reported that adult mesenchymal stem cells (MSCs) tend to gradually lose their stem cell characteristics in vitro when placed outside their niche environment. They subsequently undergo spontaneous differentiation towards mesenchymal lineages after only a few passages. We observed a similar phenomenon with adult tendon stem cells (TSCs) where expression of key tendon genes such as *Scleraxis* (*Scx*), are being repressed with time in culture. We hypothesized that an environment able to restore or maintain *Scleraxis* expression could be of therapeutic interest for in vitro use and tendon cell-based therapies.

**Methods:**

TSCs were isolated from human cadaveric Achilles tendon and expanded for 4 passages. A high content imaging assay that monitored the induction of Scx protein nuclear localization was used to screen ~1000 known drugs.

**Results:**

We identified retinoic acid receptor (RAR) agonists as potent inducers of nuclear Scx in the small molecule screen. The upregulation correlated with improved maintenance of tendon stem cell properties through inhibition of spontaneous differentiation rather than the anticipated induction of tenogenic differentiation. Our results suggest that histone epigenetic modifications by RAR are driving this effect which is not likely only dependent on Scleraxis nuclear binding but also mediated through other key genes involved in stem cell self-renewal and differentiation. Furthermore, we demonstrate that the effect of RAR compounds on TSCs is reversible by revealing their multi-lineage differentiation ability upon withdrawal of the compound.

**Conclusion:**

Based on these findings, RAR agonists could provide a valid approach for maintaining TSC stemness during expansion in vitro, thus improving their regenerative potential for cell-based therapy.

**Electronic supplementary material:**

The online version of this article (doi:10.1186/s13287-016-0306-3) contains supplementary material, which is available to authorized users.

## Background

Tendon stem cells (TSCs) have been characterized in adult tendons and show similar differentiation properties to bone marrow mesenchymal stem cells (BM-MSCs) with the exception of expressing tendon specific markers [1, 2]. Previous studies on mesenchymal stem cells (MSCs) have also shown in vitro aging and spontaneous differentiation during expansion over several passages [3–5]. Similar to MSCs removed from their niche, the absence of tendon extracellular matrix (ECM) in culture interferes with TSC self-renewal and differentiation behavior. This could explain the appearance of bone, adipose, and cartilage markers upon expansion in vitro. TSC expansion for in vitro use, or potentially cell therapy still remain to be optimized in order to preserve their stem cell characteristics while preventing premature differentiation.

*Scleraxis *(*Scx*) is a basic helix–loop–helix transcription factor highly specific to tendon and has been shown to be both sufficient and necessary to promote the tendon cell fate [6–8]. Although little is known about Scx function in stem cells, TSCs isolated from adult tendons express high levels of Scx but gradually lose this expression after being cultured. *Scx* is expressed throughout development and decreases significantly during adulthood. It is known that mechanical stimulation can upregulate *Scx* expression under physiological loading both in vitro and in vivo, suggesting that it might be required for tendon homeostasis [9, 10]. A recent study also showed that overexpression of Scx in TSCs promoted better repair compared with untransduced cells when transplanted in a patellar tendon injury model [11]. Therefore, we performed a high-content imaging screen on TSCs to discover small molecules able to induce Scx nuclear protein levels in later passage TSCs when they have low nuclear signal. We hypothesized that a drug increasing Scx signaling would be beneficial in vitro for cell therapy aimed at promoting tendon regeneration and may also provide a suitable ex vivo culture environment for TSCs.

## Methods

### Ethical approval

Achilles and patellar tendons from a 55-year-old human cadaver were obtained following informed consent from the subject or the subject’s legally authorized representative by Asterand Bioscience (Asterand, Detroit, MI, USA) according to the Department of Health and Human Services regulations for the protection of human subjects (45 CFR §46.116 and §46.117) and Good Clinical Practice (ICH E6). United States Postmortem Sites, except studies being performed by Veterans Affairs (VA) investigators at VA facilities or off-site VA locations, are exempt from institutional review board review because deceased donors are not considered “human subjects” under federal regulations for live donors (CFR 45 part 46). In compliance with the federal regulation stated for postmortem tissues, no approval from an ethics committee was necessary for this study. For rat TSC isolation, Achilles tendons from Sprague Dawley rats 3–4 months old were used following approval by the Institutional Animal Care and Use Committee (IACUC-Protocol P14-357) at the Genomics institute of the Novartis Research Foundation. The experimental animals received care in compliance with the Guide for the Care and Use of Laboratory Animals.

### Isolation and expansion of TSCs

Surrounding connective tissue was removed and the tissue was washed in Hank’s Balanced Salt Solution (HBSS) and then transferred to 5 % dispase, 3 mg/ml collagenase type 1 (Worthington, Lakewood, NJ, USA) with antibiotics/antimycotics. The tissue was digested at 37 °C for 12 hours. The solution was then passed through a 50 μm cell strainer and the cells were centrifuged at 350 × *g* for 20 minutes. Pelleted cells were resuspended in growth media and plated at a density of ~750 cells/cm^2^ in MSC expansion media (Lonza, Basel, Switzerland). Cells were cultured for 7–10 days at 37 °C, 5 % CO_2_. After colony formation, the cells were trypsinized and expanded. Cell lines from both human and rat origin were able to be maintained in culture for >30 passages.

### mRNA extraction and quantitative reverse transcription-PCR analysis

mRNA was extracted using the RNeasy Plus kit (Qiagen, Germantown, MD, USA) as per the manufacturer’s protocol. cDNA was synthesized from the isolated mRNA using qScript cDNA SuperMix (Quanta Biosciences, Gaithersburg, MD, USA) as per the manufacturer’s protocol. cDNA and primers were transferred using the Echo liquid handler system (Labcyte, Sunnyvale, CA, USA). Quantitative PCR was performed using SYBR Green (Roche, Indianapolis, IN, USA) in a 5 ul reaction and analyzed with the Roche 480 Lightcycler. Raw c(t) values were converted to 2^ΔΔc(t)^ for comparison between samples. The average of three different housekeeping genes was used as calibrators for the experiment: GADPH (metabolic), 36B4 (ribosomal), and beta-actin (cytoskeleton). Three biological replicates were used and standard deviation (SD) was calculated for each condition. For primer sequences, see Additional file 1: Table S1.

### Immunocytochemistry and high-content imaging

Cells were plated at different densities and grown for the indicated amounts of time. Cells were then fixed with 4 % paraformaldehyde, electron microscopy grade (Electron Microscopy Sciences, Hatfield, PA, USA), for 20–30 minutes at room temperature. Permeabilization and blocking was performed with 3 % bovine serum albumin (Sigma, St-Louis, MO, USA) and 0.2 % Triton X-100 (Sigma) in phosphate-buffered saline (PBS) for 1 hour at room temperature. Primary antibodies were diluted in blocking buffer and stained overnight at 4 °C. Primary antibodies against the following proteins were used: anti-SCX (Abgent, San Diego, CA, USA), anti-Oct4 (Reprocell Inc., Boston, MA, USA), and anti-aggrecan (Millipore, Billerica, MA, USA). After three washes with PBS, cells were incubated with Alexa-Fluor 488 (Life Technologies, Carlsbad, CA, USA) conjugated antibody and Hoechst 33342 dye (Life Technologies) for 1 hour at room temperature. After three washes with PBS, cells were then imaged.

In addition, cells treated with CD1530 (Tocris Bioscience, Bristol, UK), all-trans retinoic acid (Tocris Bioscience, Bristol, UK), CD2665 (Tocris Bioscience), transforming growth factor beta-2 (TGFβ2; R&D Systems, Minneapolis, MN, USA), BIX-01294 (Sigma) and C646 (Sigma) were stained using the same method. High-content imaging was performed in an ImageXpress Ultra Confocal System (Molecular Devices, Sunnyvale, CA, USA) and staining for Scx, aggrecan, and nuclei was analyzed using MetaXpress 5.0 software (Molecular Devices).

### Differentiation assays

Cells were plated in MSC media (Lonza) at 80 % confluence into six-well dishes and incubated for 8–12 hours to allow cell attachment. After the cells were attached, the media were changed to the respective differentiation cocktails ± 100 nM tazarotene (Sigma) for 14 days in vitro (DIV). Commercially available differentiation cocktails used were StemPro® Adipogenesis, Osteogenesis, and Chondrogenesis Differentiation Kits (Life Technologies). After 14 days, cells were fixed with 4 % paraformaldehyde for 30 minutes and stained for lineage specific markers. Akaline phosphatase activity in osteoblasts was revealed using Fast Blue RR (Sigma). Adipocytes were stained for lipid accumulation with LipidTOX-Green (Life Technologies). After fixation, cells were incubated with PBS containing LipidTOX for 1 hour and then imaged. Chondrocytes were examined for aggrecan accumulation using the immunofluorescence protocol already described.

### Western blots

Cells were plated at confluence grown in the presence or absence of 100 nM tazarotene for 4 days. Cells were harvested by scraping and were pelleted before extraction of total crude protein or nuclear and cytoplasmic protein fractions (NE-PER Reagents, Thermo Scientific, Waltham, MA, USA). Proteins were run on 4–12 % Bis-Tris Gels and transferred as recommended for Polyvinylidene difluoride membrane (Life Technologies). Protein blots were blocked with 3 % ECL Prime blocking buffer for 1 hour. All subsequent antibody incubations were performed in 3 % ECL Prime blocking buffer. Antibodies used were anti-SCXA (Abgent, San Diego, CA, USA) and anti-rabbit horseradish peroxidase (GE Healthcare, Chicaco, IL, USA). The signal was developed using SuperSignal West Pico substrate (Thermo Scientific, Waltham, MA, USA) and visualized using the ChemiDoc MP Imaging System (Bio-Rad, Hercules, CA, USA).

### Statistical analysis

Dose–response curves and statistical analysis were done using Graphpad Prism software (San Diego, CA, USA). To determine statistical significance between treatments, an unpaired, two-tailed Student’s *t* test was used. For all graphs, data are presented as mean ± SD. *p* <0.05 was considered significant. 

## Results

### A small low molecular weight screen to identify Scx modulators

**Fig. 1:**
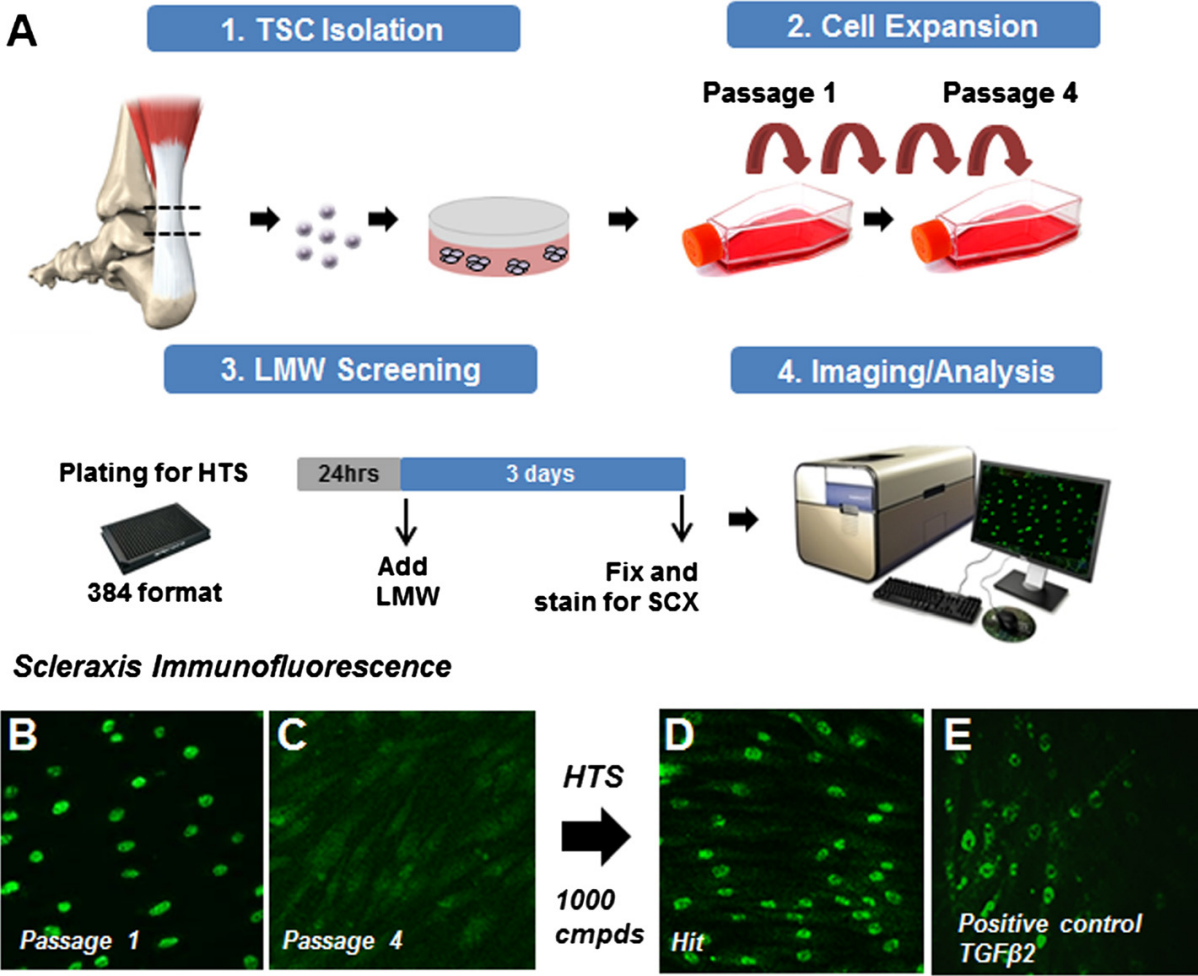
A small LMW screen designed to identify Scx upregulators using high-content imaging **a**. Human TSCs lose nuclear Scx nuclear localization with passages **b**, **c**. Hits were identified as strong Scx inducers by displaying higher levels compared with our positive control, TGFβ2 **d**, **e**. *LMW* low molecular weight, *Scx* scleraxis, *TGFβ2* transforming growth factor beta-2, *TSC* tendon stem cell

TSCs were isolated from human Achilles cadaveric tendons and plated at low density for colony formation (Fig. 1a). As reported previously, colonies were observed following 7–10 days in culture and displayed heterogeneity in size and density but produced a morphologically homogeneous population of polygonal shaped cells upon expansion. Scx immunofluorescence on early passage cells revealed strong nuclear immunoreactivity while nuclear localization was gradually lost upon expansion (Fig. 1b, c). The Scx antibody specificity was confirmed using a construct overexpressing human SCX protein in 293 cells which are negative for SCX expression, and their TSC identity was confirmed using Oct4 staining (Additional file 2: Figure S1). The decrease in nuclear localization upon culturing was used to design a low molecular weight (LMW) screen (~1000 different known drugs) to identify Scx inducers. Cells were expanded up to passage 4, a time when the vast majority of cells have low nuclear localized Scx protein, and plated in a 384-well format for high-throughput screening. The following day, the cells were stimulated with the small molecules. Cells were then grown for an additional 3 days, and then fixed and stained for Scx. Using high-content imaging, each individual well was visualized and analyzed using an algorithm that quantifies Scx nuclear translocation. TGFβ2, already known to induce Scx expression in TSCs, was used as a positive control in our screen (Fig. 1d, e).

### RAR agonists induce strong SCX nuclear localization in human TSCs

**Fig. 2:**
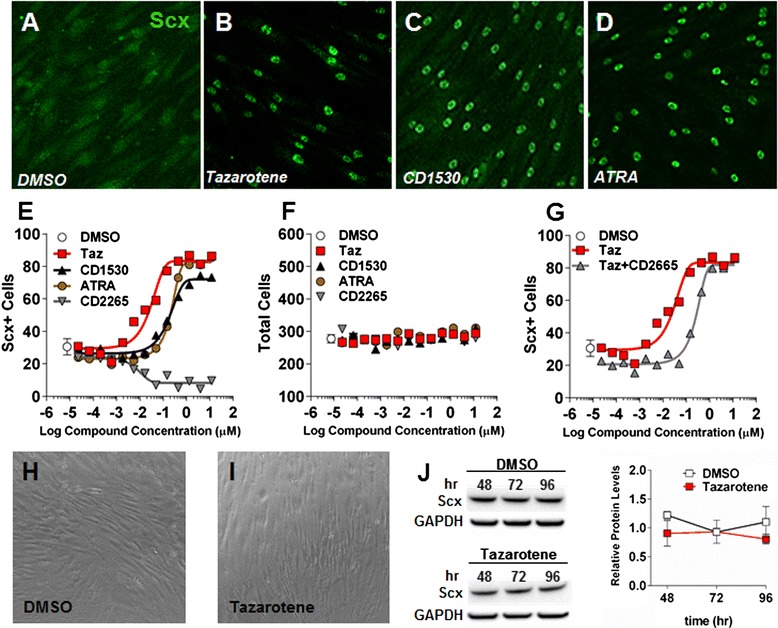
RAR agonists induce SCX nuclear localization in human TSCs **a**–**d**. The effect was observed in a dose–response manner and EC_50_ curves could be generated from a SCX nuclear localization algorithm looking at nuclear translocation and corresponding cell counts for different RAR agonists (tazarotene, CD1530, retinoic acid) and an antagonist (CD2665) **e**, **f**. Co-treatment with the RARγ antagonist CD2665 (1 μM) was able to induce a shift in the tazarotene EC_50_ curve **g**. Phase-contrast images showing different cell morphologies between control and tazarotene-treated cells **h**, **i**. Western blot quantification of total SCX protein remaining unchanged following treatment with tazarotene ***j*** *ATRA* all-trans retinoic acid, *DMSO* dimethyl sulfoxide, *hr* hours, Scx scleraxis, *Taz* tazarotene

Tazarotene, a RARβ and RARγ agonist, was identified as a strong hit in our LMW screen due to intense nuclear staining observed in treated cells (Fig. 2a, b). Further reconfirmation was carried out using two other molecules that also act as RAR agonists (CD1530 and ATRA). Both RAR agonists recapitulated the effect seen with tazarotene and showed expected half-maximal effective concentration which induces a response (EC_50_) values for such compounds (Fig. 2c–e). Interestingly, RAR antagonist CD2665 was able to reduce SCX to levels lower than dimethyl sulfoxide (DMSO) controls, suggesting that endogenous RAR activity might play a role in SCX maintenance (Fig. 2e). Cellular analysis revealed no decrease in total cell number or obvious change in morphology with any of the compounds (up to 10 μM), confirming that the nuclear localization observed was not caused by nonspecific cytotoxicity (i.e., rounding of the cells) (Fig. 2f, h, i). When added in combination, CD2665 was also able to induce a shift in the tazarotene EC_50_ curve, confirming RAR as the target (Fig. 2g). Although human cells treated with tazarotene exhibited an increase in nuclear Scx protein, the total protein levels remained unchanged, suggesting activation through nuclear translocation rather than general protein increase (Fig. 2j). We also observed a similar phenotype with TSCs isolated from human patellar tendon, suggesting the effect is not unique for Achilles TSCs but is also conserved in TSCs isolated from other tendons (Additional file 3: Figure S2).

### Tazarotene can maintain TSC identity and block differentiation into different mesenchymal lineages

Because of the limited availability of human cells at early passage, the differentiation experiments and further characterization were performed using rat TSCs isolated using an identical protocol. The increase in the nuclear Scx protein following stimulation with tazarotene was confirmed in rat TSCs by western blot using a different Scx commercial antibody that cross-reacts with rat scleraxis protein (Additional file 4: Figure S3). Tazarotene, which was the most potent RAR agonist tested in our assay, was used at 100 nM for subsequent experiments because this concentration gives close to 100 % efficacy at inducing Scx nuclear localization (Fig. 2e).

**Fig. 3:**
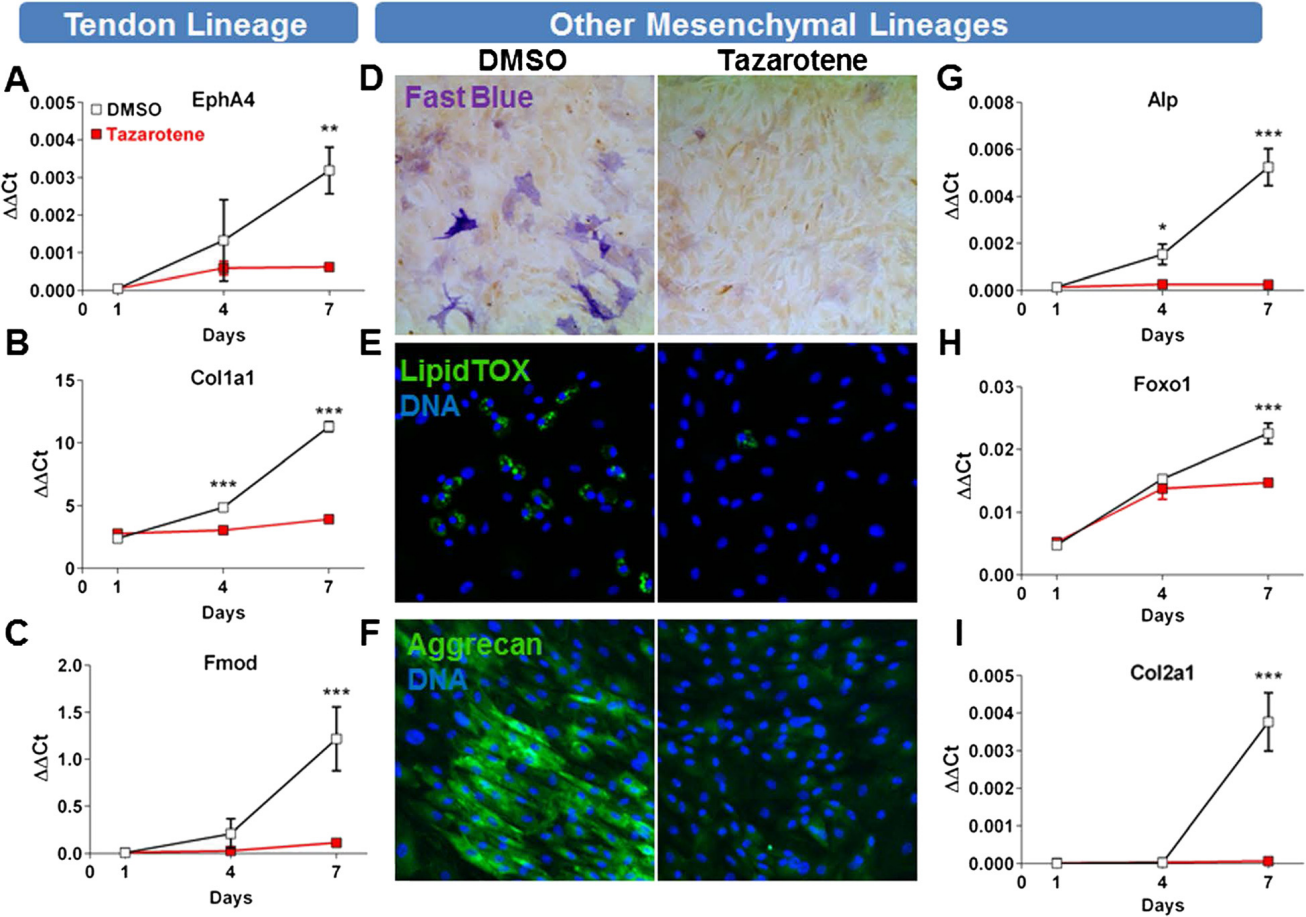
RAR agonists can prevent tenogenic differentiation and other instructed mesenchymal lineages in rat ATSCs (passage 6) following long-term differentiation in confluent conditions **a**–**i**. Stimulation with osteogenic **d**, **g**, adipogenic **e**, **h**, and chondrogenic **f**, **i** media caused a time-dependent increase in alkaline phosphatase **d**, **g**, LipidTOX-Green/*Foxo1* **e**, **h** and Aggrecan/*Col2a1* **f**, **i** respectively. Addition of tazarotene to the induction media causes a decrease in all differentiation markers **a**–**i**. Values are given as the mean ± SD, *n* = 3. *p <0.05, **p <0.005, ***p <0.001. *DMSO* dimethyl sulfoxide

**Fig. 4:**
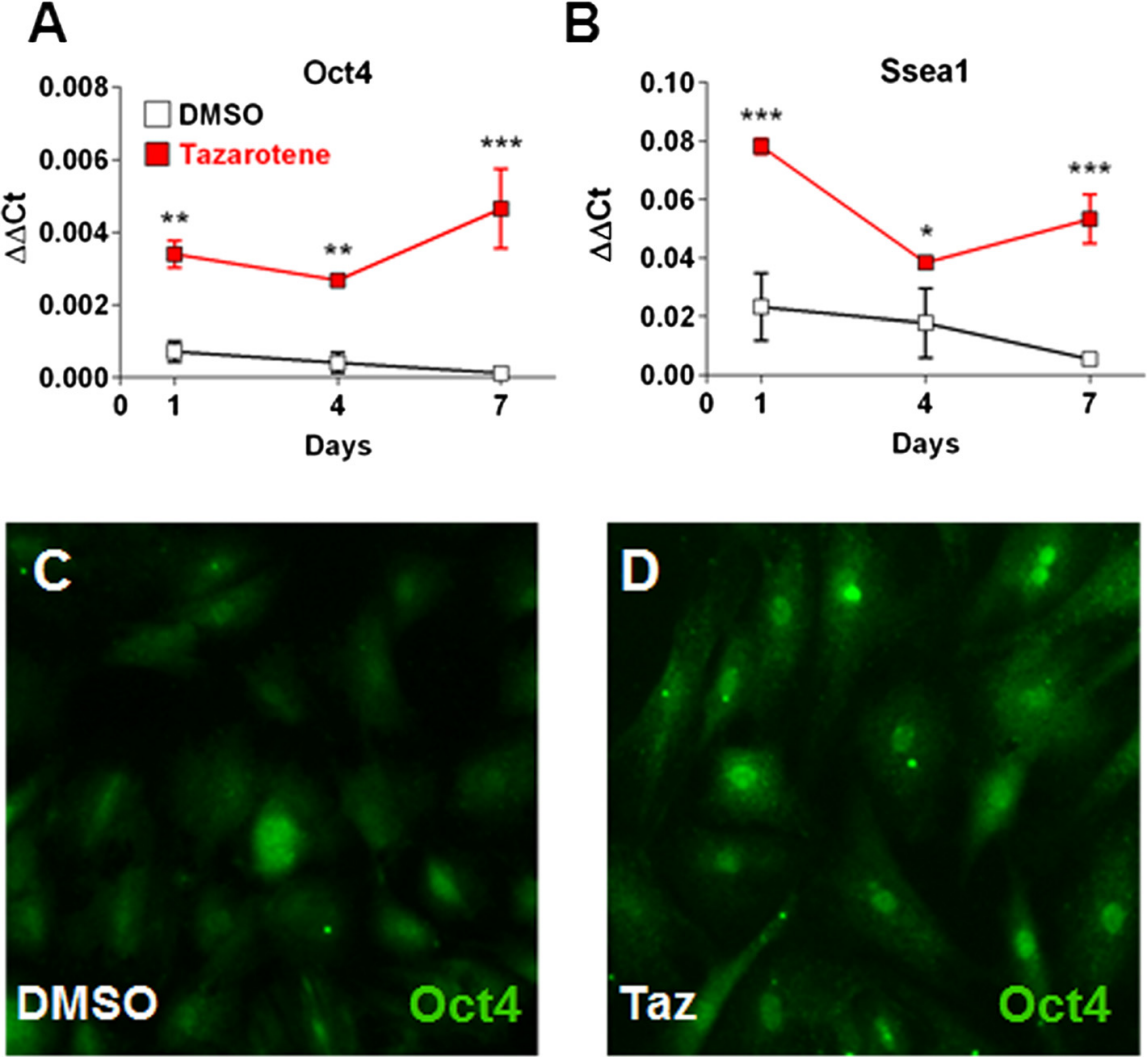
Stem cell marker maintenance following treatment with tazarotene **a**–**d**. *Oct4* and *Ssea-1* mRNA levels are higher in tazarotene-treated cells compared with DMSO control **a**, **b**. *Oct4* immunofluorescence staining after 4 days confirms higher nuclear levels in tazarotene-treated cells **c**, **d**. Values are given as the mean ± SD, *n* = 3. *p <0.05, **p <0.005, ***p <0.001. *DMSO* dimethyl sulfoxide, *Taz* tazarotene

Even though *Scx* is expressed in TSCs, its increase in expression has also been associated with tenogenic differentiation both in vitro and in vivo [6, 7, 12–14]. To determine whether tazarotene could potentially induce tenogenesis, a differentiation time-course assay was performed in vitro. Following confluency, a time-dependent increase in tendon gene expression (*EphA4*, *Col1a1*, and *Fmod*) was observed, reaching a maximum at 7 days. However, cells treated with tazarotene did not show a greater increase in expression and instead displayed mRNA levels at day 7 similar to those seen at day 1, suggesting that tazarotene is perhaps maintaining the cells in an undifferentiated state (Fig. 3a–c). TSCs have the ability to differentiate into each of the major MSC lineages when stimulated in the appropriate conditions. When treated with osteogenic, adipogenic, or chondrogenic induction cocktails, the cells responded as predicted—shown by specific histological staining and quantitative PCR for each lineage. Osteogenic induction media caused an upregulation of the alkaline phosphatase (*Alp*) messenger RNA as well as by fast blue staining (Fig. 3d, g). Adipogenic induction media caused an upregulation of* Foxo1 *and lipid accumulation shown with LipidTOX-Green staining, while chondrogenic media increased expression of both aggrecan and collagen type 2 (Fig. 3e, f, h, i). Each of the lineages also took on the characteristic morphology of their respective lineage. Addition of tazarotene was able to suppress the induction of differentiation for all lineages, as seen by negative staining and low mRNA levels for up to 7 days (Fig. 3a–i). Inversely, treatment with tazarotene could maintain stem cell markers (*Oct4* and *Ssea-1*) for up to 7 days, confirming the inhibition of differentiation and maintenance of a stem cell phenotype (Fig. 4a–d).

### Tazarotene prevents spontaneous differentiation during expansion

**Fig. 5:**
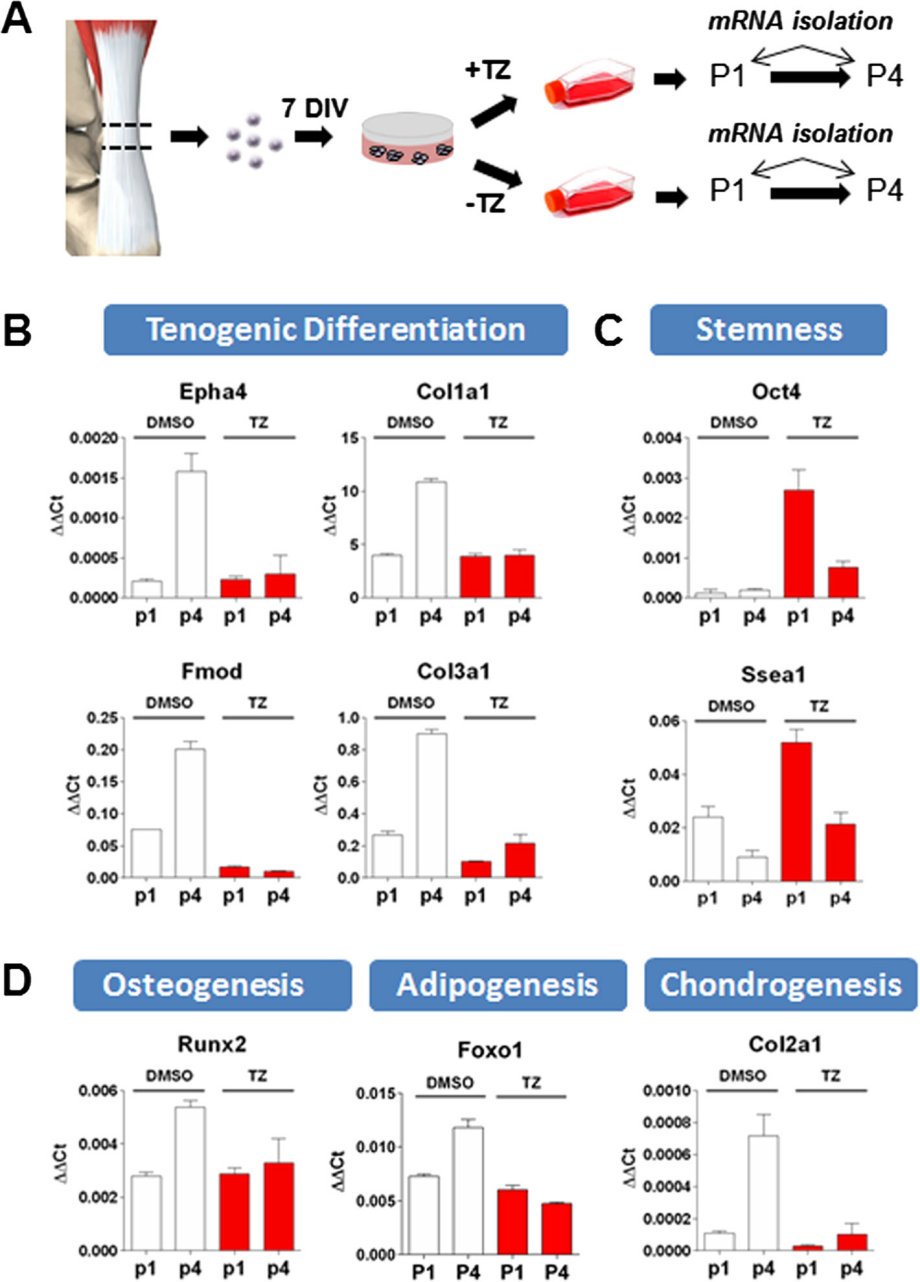
Tazarotene prevents spontaneous differentiation arising with extended culture time. TSCs were isolated from adult rat Achilles tendons and plated at low density for colony formation before being trypsinized and grown with or without tazarotene (*TZ*). Messenger RNA was collected at early (*P1*) and late passages (*P4*) for gene expression analysis **a**. In control conditions, several genes typical of TSC differentiation towards multiple lineages were found upregulated in late passage cells (P1 vs P4), confirming spontaneous differentiation during in vitro cell expansion **b**, **d**. Addition of tazarotene to the culture media was able to preserve TSC’s stemness as seen by cells from passage 4 having similar expression levels to those of passage 1 **c**. Values are given as the mean ± SD, *n* = 3

It is known that MSCs from different origins change progressively in culture, with some cells undergoing spontaneous or premature differentiation during successive passages [3–5]. Since TSCs are relatively similar to MSCs, we hypothesized that the expansion of cells in the presence of tazarotene would prevent this process and preserve their stem cell characteristics. To test this hypothesis, TSCs were isolated from naïve rat Achilles tendons and plated at clonal density for 7 days. Colonies were then trypsinized and plated at subconfluency with or without tazarotene. Every 3–4 days, the same of amount of cells from either condition were replated and treated for four consecutive passages. mRNA was harvested at the first and fourth passages for gene expression analysis (Fig. 5a). As hypothesized, cells grown in the absence of tazarotene showed an increase in several markers associated with differentiation towards multiple mesenchymal lineages. Gene expression level comparison from passage 1 with passage 4 revealed significant upregulation of tenogenic (*EphA4, Col1a1, Col3a1, Bgn*), osteogenic (*Runx2*), adipogenic (*Foxo1*), and chondrogenic (*Col2a1*) genes (Fig. 5b, d). Inversely, stem cell marker expression (*Oct4 *and *Ssea-1*) was higher in tazarotene expanded cells (Fig. 5c). The preservation of stem cell marker expression could be observed over several passages (Additional file 5: Figure S4). These findings suggest tazarotene could maintain the TSC phenotype and prevent spontaneous differentiation during cell expansion.

### Inhibition of differentiation by tazarotene is reversible upon withdrawal

**Fig. 6:**
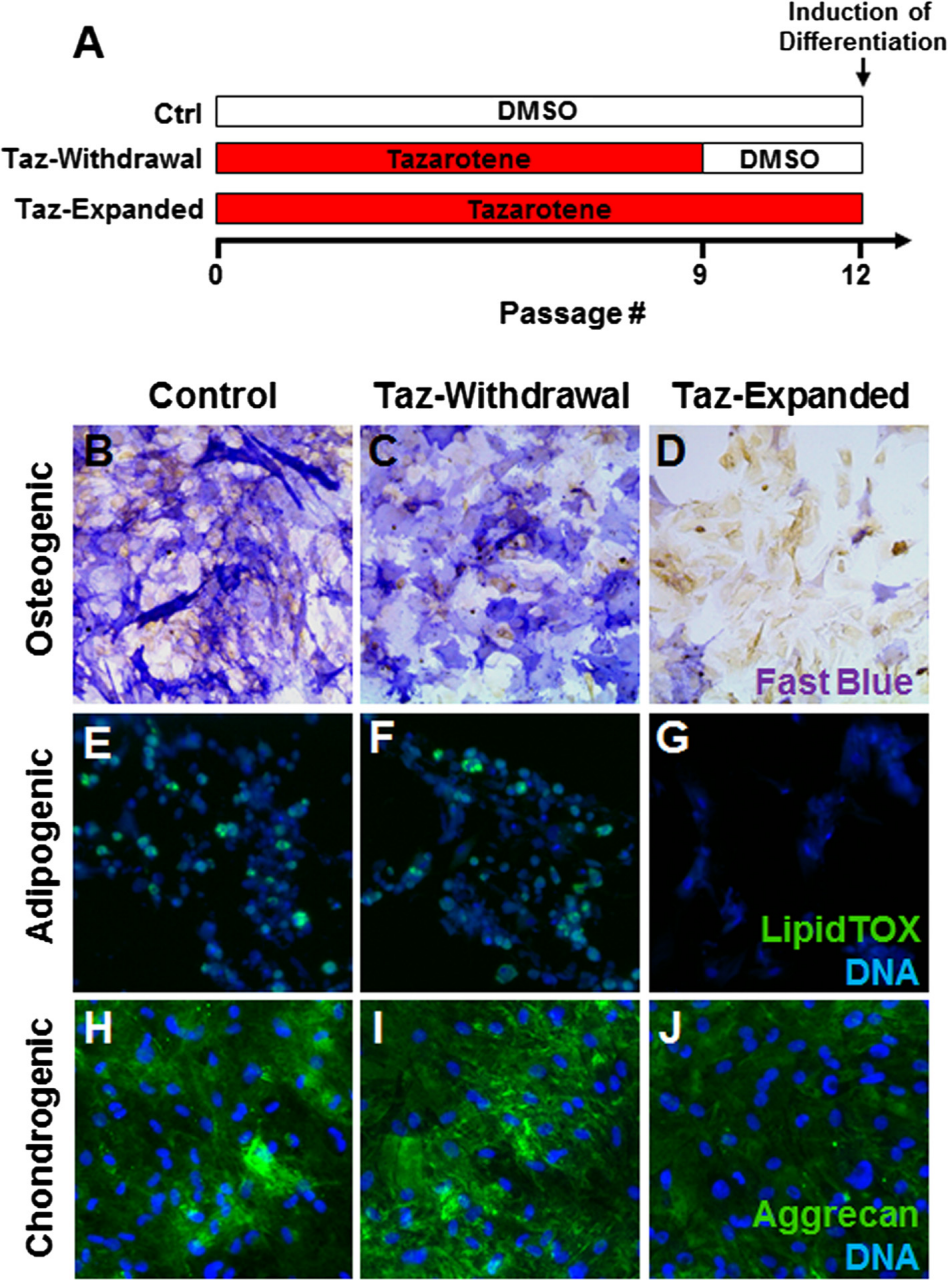
Differentiation potential of tazarotene-expanded TSCs is conserved upon withdrawal. Tazarotene-expanded cells from passage 9 were cultured with or without the compound for three further passages and induced to differentiate towards different mesenchymal lineages **a**. Both conditions were equally able to differentiate into osteocytes, adipocytes, and chondrocytes as seen by upregulation of alkaline phosphatase, LipidTOX, and aggrecan staining **b**, **c**, **e**, **f**, **h**, **i**. However, TSCs at the same passage cultured in a constant presence of tazarotene did not differentiate **d**, **g**, **j**. *DMSO* dimethyl sulfoxide, *Taz* tazarotene

Because tazarotene appeared to prevent differentiation in subconfluent conditions, we aimed to determine whether this blockade was reversible upon removal of the compound. TSCs grown in the presence of tazarotene for nine passages were further expanded with or without the compound for an additional three passages and induced to differentiate into different lineages (Fig. 6a). Cells from both of these conditions were then plated in the absence of compound and induced to differentiate into osteocytes, adipocytes, or chondrocytes for 14 DIV while using the untreated passage 12 cells as a control. Control cells displayed alkaline phosphatase reactivity in addition to LipidTOX-Green and aggrecan-positive staining when stimulated with osteogenic, adipogenic, and chondrogenic media respectively (Fig. 6b, e, h). Similar to the control cells, withdrawal of the compound for three consecutive passages was sufficient to restore multiple lineage differentiation capabilities to the cells (Fig. 6c, f, i). Cells expanded in the presence of tazarotene until the same passage number (passage 12) were not able to differentiate despite the absence of the compound in the induction media, suggesting that additional cell divisions following withdrawal might be required to fully restore their competence to differentiate (Fig. 5d, g, j). We also confirmed that tazarotene treatment does not have a major impact on cell proliferation at efficacious doses. The population doubling time at concentrations between 20 and 500 nM did not show significant difference compared with untreated cells and was also unchanged following withdrawal of the compound (Additional file 6: Figure S5).

### Histone methylation appears necessary for SCX nuclear induction by tazarotene

**Fig. 7:**
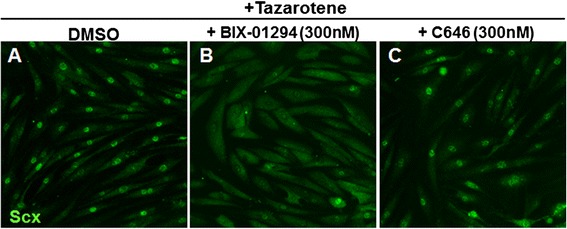
Tazarotene induces Scx nuclear localization through histone modifications. Human TSCs from passage 4 were co-treated for 4 DIV with histone methyltransferase and acetyltransferase inhibitors in the presence of tazarotene **a**–**c**. Addition of BIX-01294, a histone methyltransferase inhibitor, was able to suppress Scx nuclear localization induced by tazarotene, while C646, a histone acetyltransferase inhibitor, did not have any effect **b**, **c**. *DMSO* dimethyl sulfoxide, *Scx* scleraxis

To verify any possible epigenetic histone modifications induced by RAR, we took advantage of the Scx nuclear translocation assay and tested different histone methyltransferase and acetyltransferase inhibitors (BIX-01294 and C646, respectively) to assess whether inhibiting such modifications at the chromatin level would be sufficient to block the effect of tazarotene on Scx nuclear translocation. For this experiment, cells were plated in the presence of tazarotene while being co-treated with each inhibitor for 4 DIV. As shown previously, Scx immunofluorescence revealed strong nuclear translocation in tazarotene-treated cells (Fig. 7a). Co-treatment with C646 did not have an effect on Scx induction, whereas BIX-01294 was able to suppress the SCX nuclear localization. This indicates that histone methylation plays a role Scx nuclear binding via RAR signaling (Fig. 7b, c).

## Discussion

Using a high-content imaging screen, we were able to identify RAR agonists as potent inducers of Scx nuclear binding in human TSCs. Stimulation of RARs on stem cells from different origins have shown both pro-differentiating and anti-differentiating activities [15–18]. However, the role of RAR in TSCs has not yet been characterized. Although Scx appears to be necessary for tendon development, it remains expressed in freshly isolated TSCs from adult tendons. In our study we have shown that maintaining Scx localization in the nucleus using a RAR agonist does not necessarily induce tenogenic differentiation but is rather associated with the maintenance of the TSC phenotype, consistent with the inhibition of differentiation towards multiple mesenchymal lineages. Even though RAR agonists were identified in our screen, it does not appear that their inhibitory activity on differentiation is entirely mediated through Scx. Knockdown experiments using small interfering mRNA against *Scx* transcript did not revert the inhibition of differentiation induced by tazarotene, suggesting that additional transcription factors might be involved (Additional file 7: Figure S6).

TSCs could be cultured in presence of tazarotene for up to 20 passages with a similar population doubling time compared with untreated cells. The maintenance of stem cell marker expression levels remained similar for up to 10 passages but slowly decreased afterwards, while still remaining higher than untreated cells (Additional file 5: Figure S4). It is possible that TSCs slowly become irresponsive to tazarotene treatment following prolonged exposure. At concentrations higher than 500 nM, we could also observe a reduction in proliferation of about 50–75 %. Tazarotene is a selective RARβ and RARγ agonist but it can also bind RARα at higher concentrations [19]. Activation of RARα has been previously shown to inhibit cell proliferation in other cell types, which could explain the slow decrease in cell division we observed at higher concentrations [20]. The anti-differentiation effect of tazarotene appears to be partially conserved among different MSC types depending on their origin. We found that tazarotene has similar activity on MSCs derived from adipose tissue but has the opposite effect on MSCs isolated from bone marrow by being pro-osteogenic (data not shown). This suggests that RAR signaling might have a different role in BM-MSCs.

RARs have multiple functions at the nuclear level by interacting with retinoid X receptors and other co-repressor or activator proteins leading to epigenetic and gene expression changes [21, 22]. Stem cell homeostasis is usually maintained through these mechanisms that are highly dynamic in regulating chromatin structure as well as specific gene expression programs involved in self-renewal and differentiation [23]. In general, stem cells show a more decondensed chromatin which contributes to an open or accessible state as compared with differentiated cells [24]. The overall increased levels of histone modifications, which are commonly transcriptionally active regions, are enriched in stem cells while silenced regions are strongly reduced compared with more differentiated cells [25]. Our results suggest that RAR agonists could preserve TSC stemness through epigenetic modifications, specifically involving histone methylation. Co-treatment with a histone methyltransferase inhibitor was sufficient to block tazarotene-induced Scx nuclear localization, while an acetyltransferase inhibitor did not have any effect.

It is possible that the binding elements and/or promoter regions of Scx and other transcription factors critical for TSC identity are gradually repressed with passages due to changes in chromatin structure. Interestingly, hypoxia has been shown to preserve TSC stemness in culture and further reduce differentiation in culture conditions [26]. Another possible explanation could be that mechanical loading in vitro is necessary for maintaining TSC properties similar to what has been shown for the maintenance Scx nuclear localization [9]. We can hypothesize that expansion in normoxia conditions and/or in the absence of mechanical stimulation might induce epigenetic changes responsible for the decrease in Scx nuclear binding and spontaneous differentiation occurring during the expansion process. These changes could be driven by an overall decrease in histone methylation and could be potentially reversed using RAR agonists. DNA methylation has also been shown to prevent spontaneous differentiation of mesenchymal progenitors in culture where removing methyl groups using 5-azacytidine causes differentiation towards the osteogenic and adipogenic cell fate [27]. The direct link between RAR and Scx nuclear localization still remains to be investigated. Further epigenetic analyses on TSCs treated with RAR agonists should help elucidate this process. Additionally, analyzing the methylation status of the SCX gene as well as other key genes involved in self-renewal and differentiation could reveal more details on how RAR agonists affect the epigenetics of TSCs.

## Conclusion

Cell-based therapies using adult stem cells harvested from the target tissue represent a potential strategy to address the unmet medical need for tendon regeneration [28–30]. Recent advances in such an approach show promising results but additional work is essential to understand how best to expand and prepare these cells for treatment and avoid premature differentiation during the expansion process. These non-tendon committed progenitors could potentially engraft following transplantation to a site of tendon injury and interfere with tendon healing and biomechanical properties by producing the wrong ECM in vivo. Tendon calcification and cartilage-like differentiation has been observed in clinical samples of tendinopathy, reinforcing the idea that engrafting such non-tendon progenitors would be detrimental [31]. In this study, we demonstrated that TSCs, like MSCs, undergo spontaneous differentiation in vitro and that addition of a RAR agonist to the culture media was able to prevent this process. In vivo studies comparing TSCs expanded with or without a RAR agonist should reinforce the use of molecules that preserve their stem cell characteristics during the expansion phase.

## Additional files

Additional file 1: is Table S1 presenting the quantitative PCR primer list. (TIF 2735 kb) Additional file 2: is Figure S1 showing Scx antibody specificity and TSC identity. Transduced 293T cells using a lentivirus overexpressing Scx with Zsgreen reporter show clear nuclear staining for Scx protein while control lentiviral vector did not show any staining **A**, **B**. Only TSCs but not tenocytes from the same tendon tissue were positive for Oct4, confirming that our protocol for stem cell isolation was successful **C**, **D**. (TIF 4337 kb) Additional file 3: is Figure S2 showing that tazarotene treatment increases nuclear Scx translocation in human patellar TSCs. TSCs isolated from human patellar tendon lose nuclear Scx localization with passages **A**, **C**. Treatment with tazarotene at 100 nM is able to induce Scx nuclear translocation similar to Achilles TSCs **B**, **D**, **E**. (TIF 1345 kb) Additional file 4: is Figure S3 showing that tazarotene treatment increases nuclear Scx translocation in rat Achilles TSCs. Western blot showing enrichment in Scx protein in the nuclear extract **A**, **B**. (TIF 734 kb) Additional file 5: is Figure S4 showing that tazarotene can maintain stem cell marker expression in TSCs for up to 17 passages. mRNA levels of both Oct4 and Ssea1 remain high in the presence of the drug for up to 17 passages **A**. Withdrawal of the compound at passage 8 is followed by a rapid decrease of both Oct4 and Ssea1 which is already visible at passage 10 **B**. (TIF 719 kb) Additional file 6: is Figure S5 showing TSC expansion in the presence of tazarotene does not affect the population doubling time. A growth curve for up to passage 17 was performed and did not show statistical difference with control DMSO-treated cells for concentrations between 20 and 500 nM **A**. Withdrawal of the compound at passage 8 is not followed by a change in population doubling time **B**. (TIF 811 kb) Additional file 7: is Figure S6 showing that Scx siRNA knockdown does not suppress the inhibitory effect of tazarotene on osteogenic differentiation. TSCs were transfected with siRNA against Scx or with a siRNA control and were induced to differentiate towards the osteogenic lineage and visualized by alkaline phosphatase staining **A**, **B**. The inhibition of osteogenic differentiation in presence of tazarotene was not blocked following Scx siRNA knockdown **C**, **D**. (TIF 3491 kb)

## Abbreviations

ATRA: All-trans retinoic acid
BM-MSC: Bone marrow mesenchymal stem cell
DIV: Days in vitro
DMSO: Dimethyl sulfoxide
EC_50_: Half-maximal effective concentration which induces a response
ECM: Extracellular matrix
HBSS: Hank’s Balanced Salt Solution
LMW: Low molecular weight
MSC: Mesenchymal stem cell
PBS: Phosphate-buffered saline
RAR: Retinoic acid receptor
Scx: Scleraxis
TGFβ2: Transforming growth factor beta-2
TSC: Tendon stem cell
