# Erratum to: Retinoic acid receptor signaling preserves tendon stem cell characteristics and prevents spontaneous differentiation in vitro

**DOI:** 10.1186/s13287-016-0327-y

**Published:** 2016-04-22

**Authors:** Stuart Webb, Chase Gabrelow, James Pierce, Edwin Gibb, Jimmy Elliott

**Affiliations:** Genomics Institute of the Novartis Research Foundation, 10675 John Jay Hopkins Drive, San Diego, CA 92121 USA

Following publication of the original article in Stem Cell Research & Therapy [1], we would like to alert the reader that the word “vitrox” in the title is incorrect and should read as “vitro”.

The corrected title reads: Retinoic acid receptor signaling preserves tendon stem cell characteristics and prevents spontaneous differentiation in vitro.

The original article has been updated with the corrected title. The publisher apologizes for the inconvenience this may have caused.
