# Supplementary figures and images for: Retinoic acid receptor signaling preserves tendon stem cell characteristics and prevents spontaneous differentiation in vitrox
Source: Stem Cell Res Ther. 2016 Mar 22;7:45. doi: 10.1186/s13287-016-0306-3 (PMC4802591; doi:10.1186/s13287-016-0306-3)

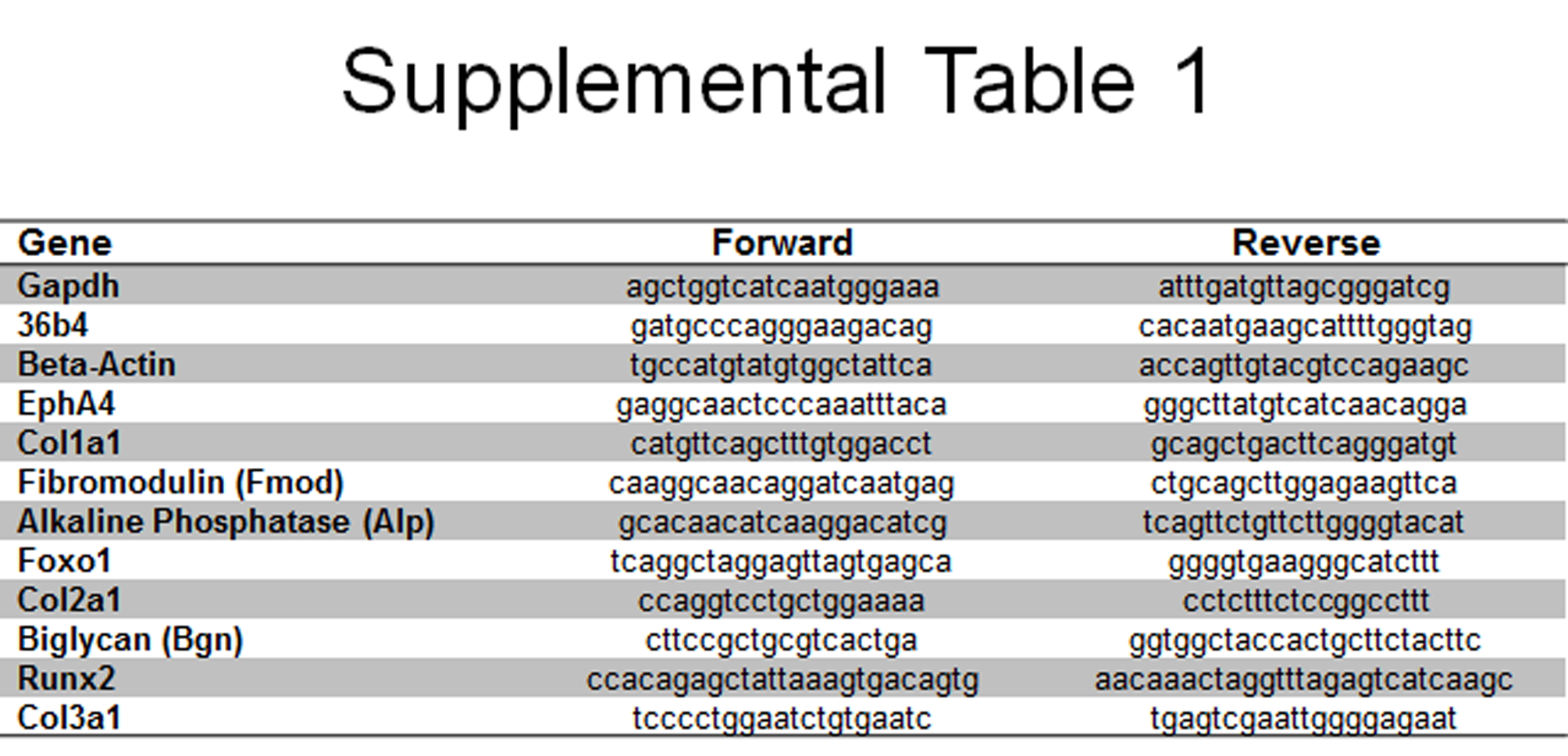

Supplement: Additional file 1: — is Table S1 presenting the quantitative PCR primer list. (TIF 2735 kb) [file 13287_2016_306_MOESM1_ESM.tif]

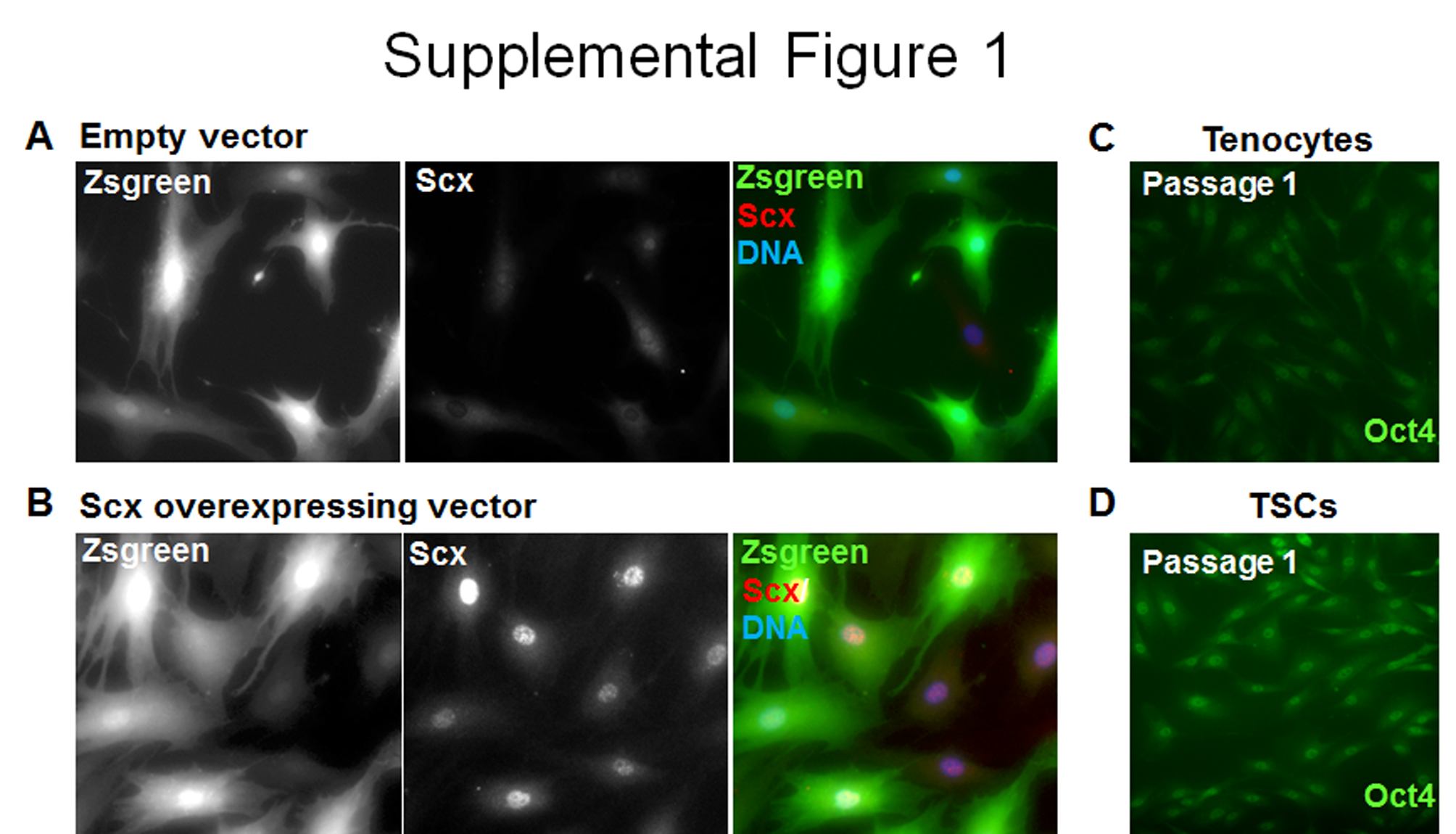

Supplement: Additional file 2: — is Figure S1 showing Scx antibody specificity and TSC identity. Transduced 293T cells using a lentivirus overexpressing Scx with Zsgreen reporter show clear nuclear staining for Scx protein while control lentiviral vector did not show any staining A, B. Only TSCs but not tenocytes from the same tendon tissue were positive for Oct4, confirming that our protocol for stem cell isolation was successful C, D. (TIF 4337 kb) [file 13287_2016_306_MOESM2_ESM.tif]

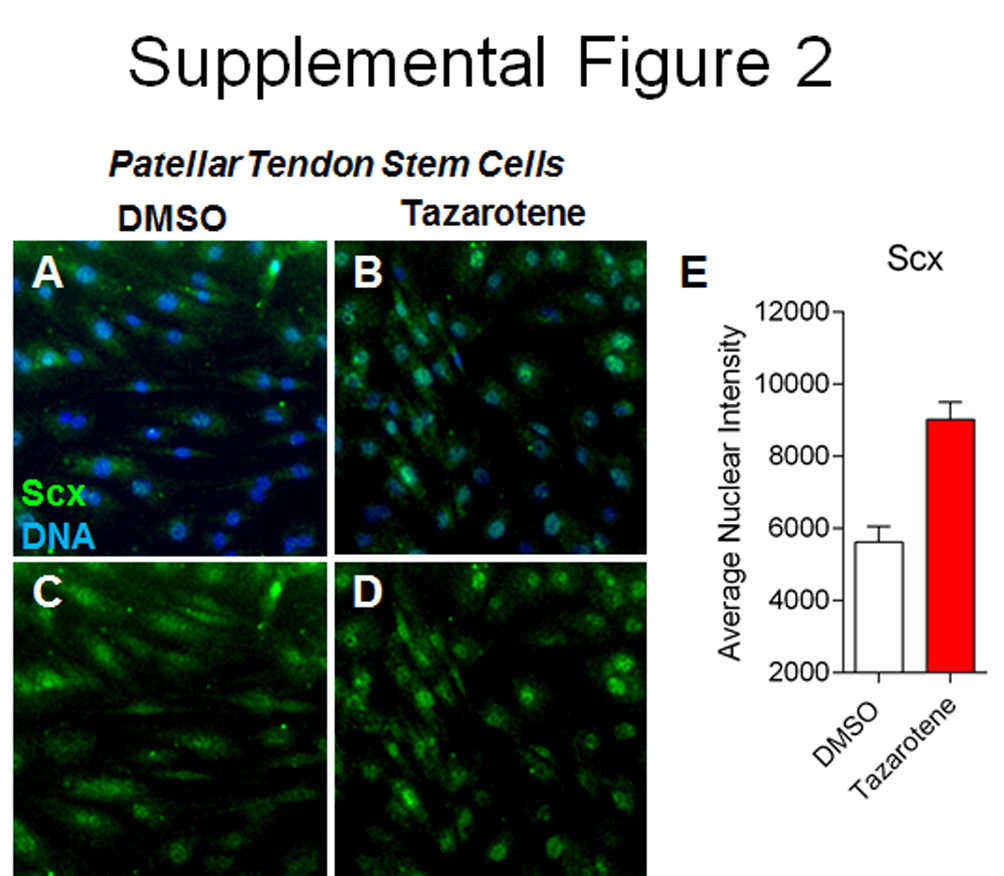

Supplement: Additional file 3: — is Figure S2 showing that tazarotene treatment increases nuclear Scx translocation in human patellar TSCs. TSCs isolated from human patellar tendon lose nuclear Scx localization with passages A, C. Treatment with tazarotene at 100 nM is able to induce Scx nuclear translocation similar to Achilles TSCs B, D, E. (TIF 1345 kb) [file 13287_2016_306_MOESM3_ESM.tif]

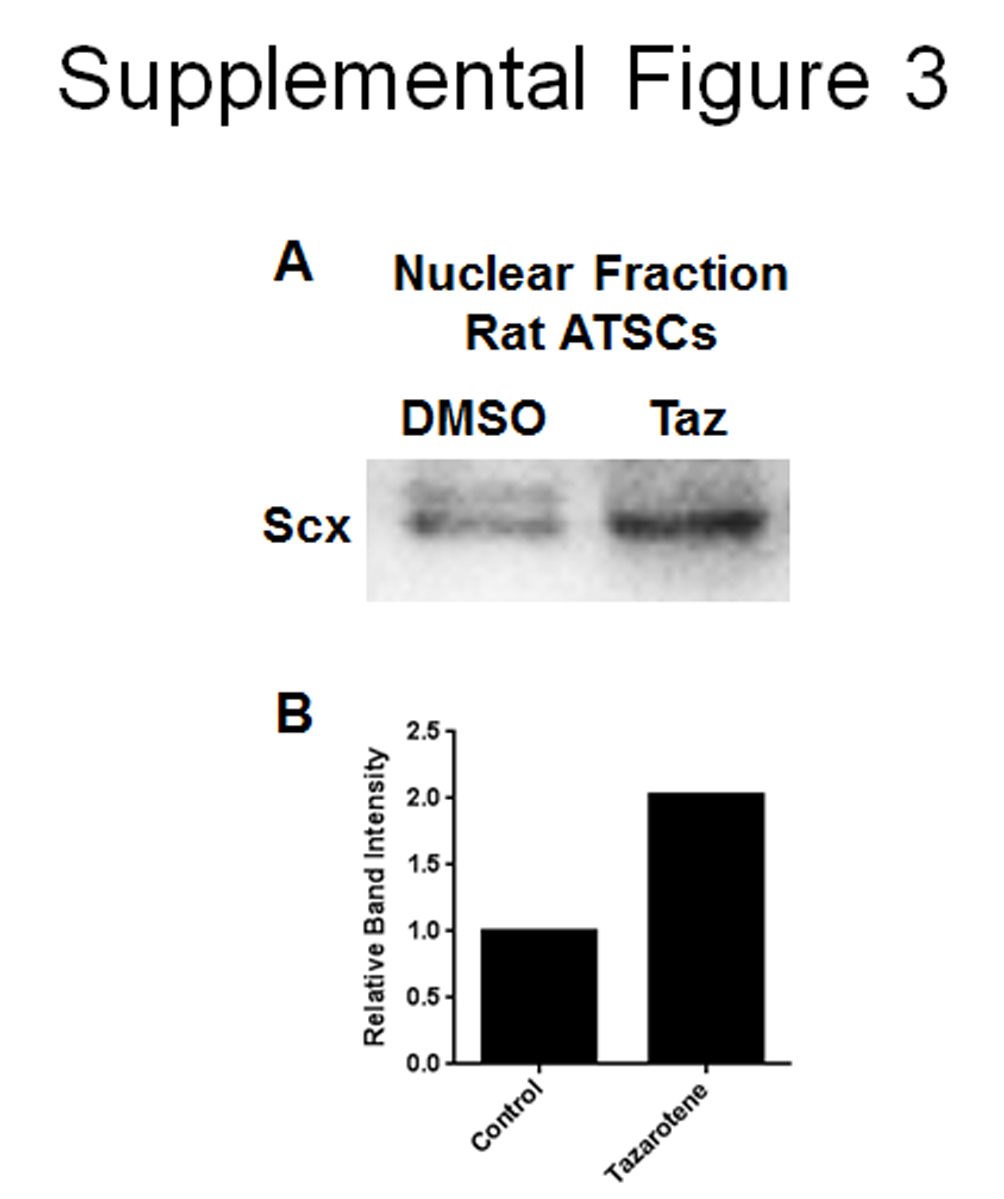

Supplement: Additional file 4: — is Figure S3 showing that tazarotene treatment increases nuclear Scx translocation in rat Achilles TSCs. Western blot showing enrichment in Scx protein in the nuclear extract A, B. (TIF 734 kb) [file 13287_2016_306_MOESM4_ESM.tif]

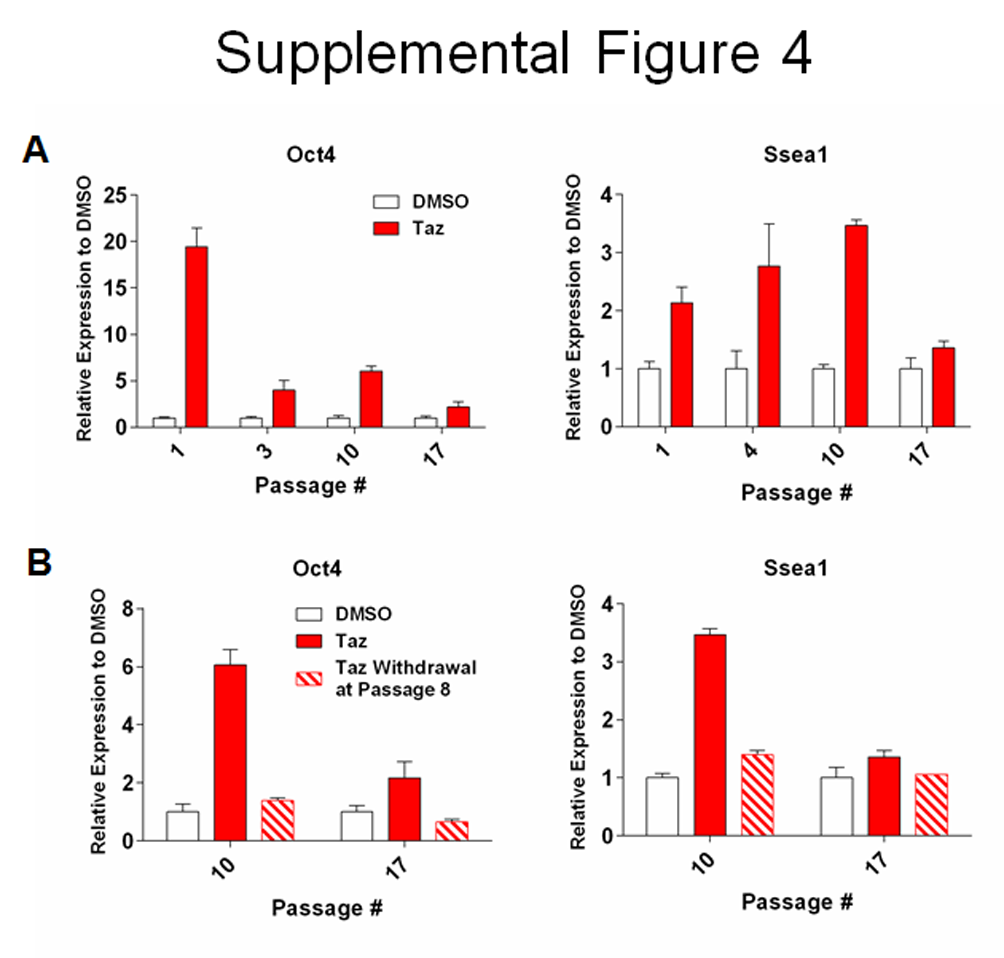

Supplement: Additional file 5: — is Figure S4 showing that tazarotene can maintain stem cell marker expression in TSCs for up to 17 passages. mRNA levels of both Oct4 and Ssea1 remain high in the presence of the drug for up to 17 passages A. Withdrawal of the compound at passage 8 is followed by a rapid decrease of both Oct4 and Ssea1 which is already visible at passage 10 B. (TIF 719 kb) [file 13287_2016_306_MOESM5_ESM.tif]

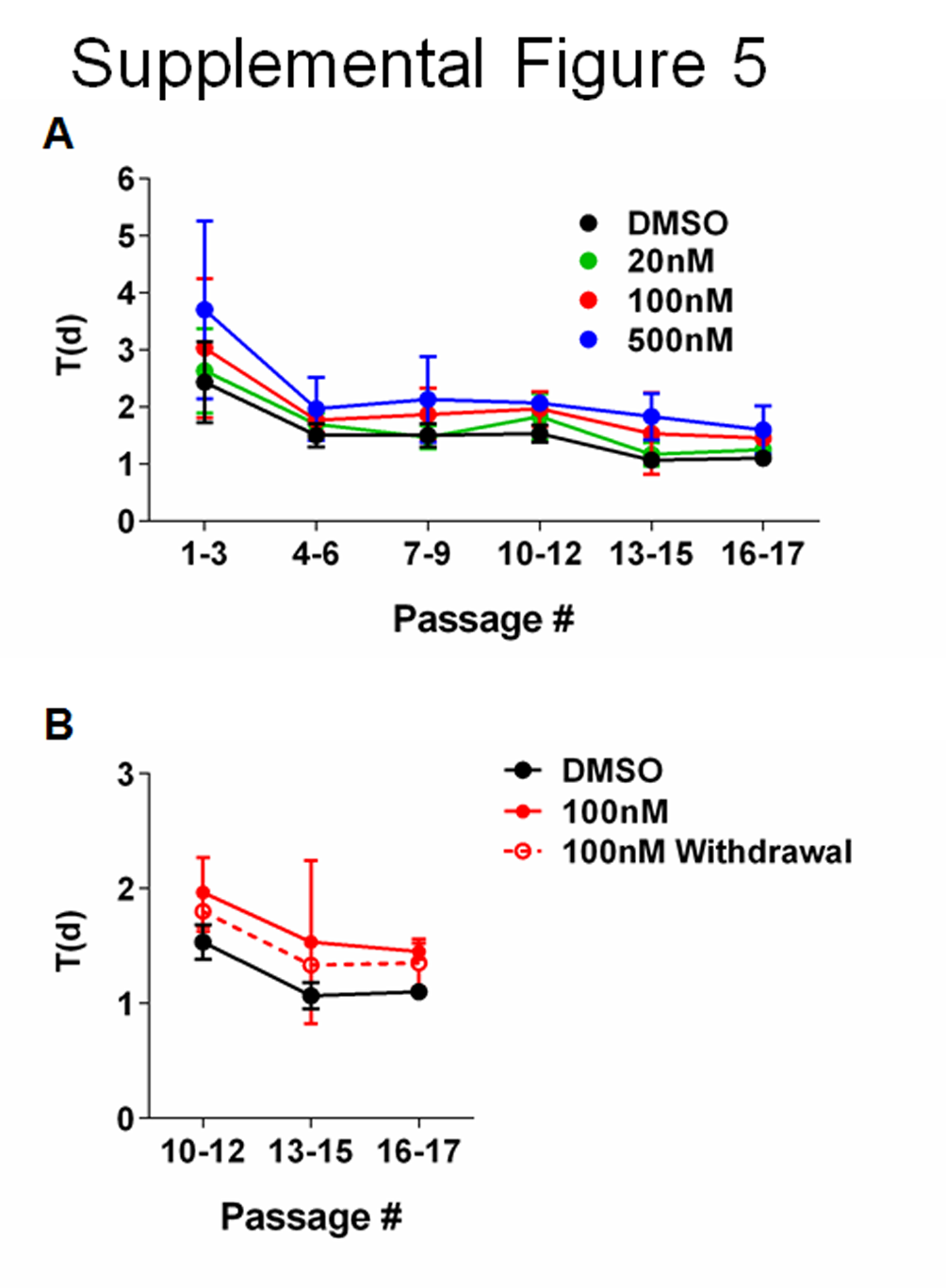

Supplement: Additional file 6: — is Figure S5 showing TSC expansion in the presence of tazarotene does not affect the population doubling time. A growth curve for up to passage 17 was performed and did not show statistical difference with control DMSO-treated cells for concentrations between 20 and 500 nM A. Withdrawal of the compound at passage 8 is not followed by a change in population doubling time B. (TIF 811 kb) [file 13287_2016_306_MOESM6_ESM.tif]

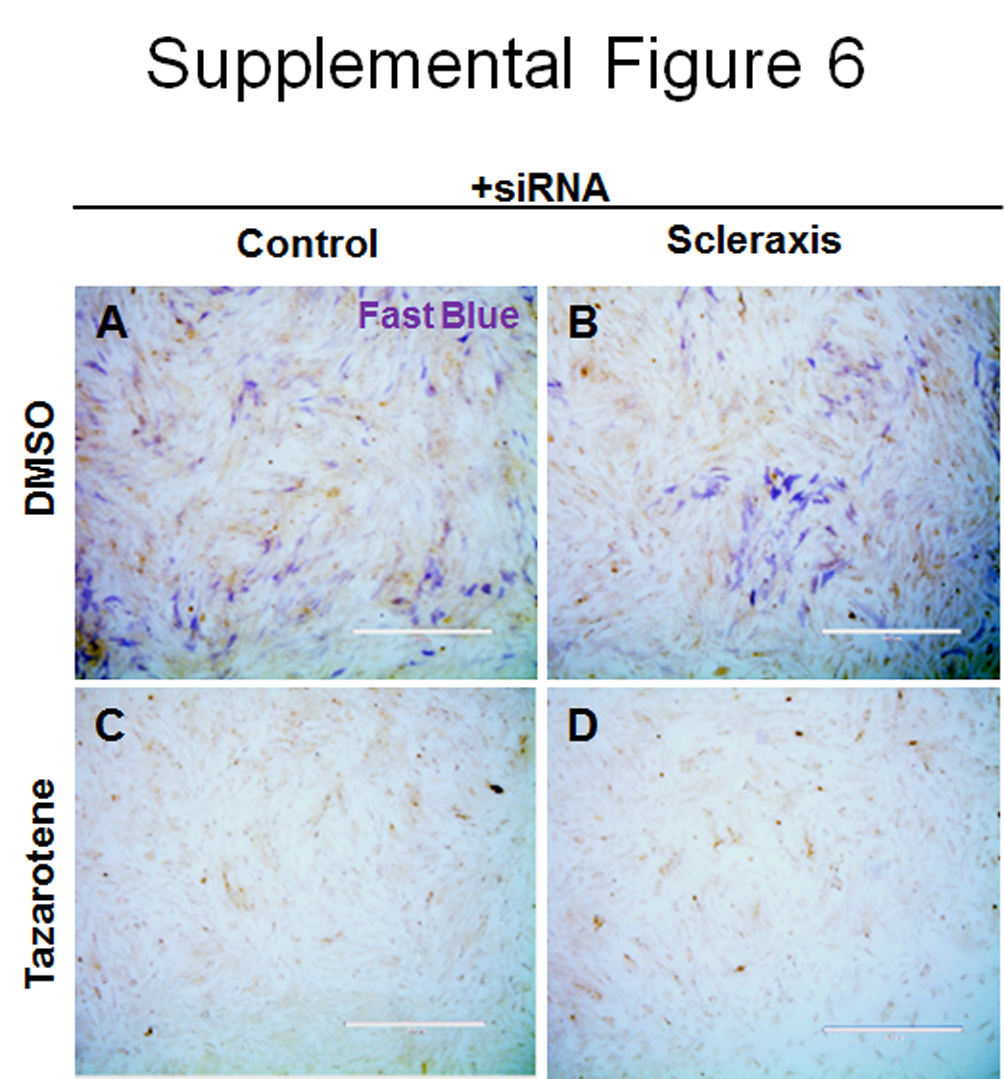

Supplement: Additional file 7: — is Figure S6 showing that Scx siRNA knockdown does not suppress the inhibitory effect of tazarotene on osteogenic differentiation. TSCs were transfected with siRNA against Scx or with a siRNA control and were induced to differentiate towards the osteogenic lineage and visualized by alkaline phosphatase staining A, B. The inhibition of osteogenic differentiation in presence of tazarotene was not blocked following Scx siRNA knockdown C, D. (TIF 3491 kb) [file 13287_2016_306_MOESM7_ESM.tif]
